# Hypoxia Regulates DPP4 Expression, Proteolytic Inactivation, and Shedding from Ovarian Cancer Cells

**DOI:** 10.3390/ijms21218110

**Published:** 2020-10-30

**Authors:** Laura R. Moffitt, Maree Bilandzic, Amy L. Wilson, Yiqian Chen, Mark D. Gorrell, Martin K. Oehler, Magdalena Plebanski, Andrew N. Stephens

**Affiliations:** 1 Department of Molecular and Translational Sciences, Monash University, Clayton, VIC 3168, Australia; laura.moffitt@monash.edu (L.R.M.); maree.bilandzic@hudson.org.au (M.B.); amy.wilson@hudson.org.au (A.L.W.); yiqian.chen@hudson.org.au (Y.C.); 2 Centre for Cancer Research, Hudson Institute of Medical Research, Clayton, VIC 3168, Australia; 3 Centenary Institute, Faculty of Medicine and Health, University of Sydney, Camperdown, NSW 2006, Australia; m.gorrell@centenary.org.au; 4 Department of Gynaecological Oncology, Royal Adelaide Hospital, Adelaide, SA 5000, Australia; martin.oehler@adelaide.edu.au; 5 Robinson Institute, University of Adelaide, Adelaide, SA 5000, Australia; 6 School of Health and Biomedical Sciences, RMIT University, Bundoora, VIC 3082, Australia; magdalena.plebanski@rmit.edu.au

**Keywords:** ovarian cancer, DPP4, hypoxia, MMP, tumour microenvironment

## Abstract

The treatment of ovarian cancer has not significantly changed in decades and it remains one of the most lethal malignancies in women. The serine protease dipeptidyl peptidase 4 (DPP4) plays key roles in metabolism and immunity, and its expression has been associated with either pro- or anti-tumour effects in multiple tumour types. In this study, we provide the first evidence that DPP4 expression and enzyme activity are uncoupled under hypoxic conditions in ovarian cancer cells. Whilst we identified strong up-regulation of *DPP4* mRNA expression under hypoxic growth, the specific activity of secreted DPP4 was paradoxically decreased. Further investigation revealed matrix metalloproteinases (MMP)-dependent inactivation and proteolytic shedding of DPP4 from the cell surface, mediated by at least MMP10 and MMP13. This is the first report of uncoupled DPP4 expression and activity in ovarian cancer cells, and suggests a previously unrecognized, cell- and tissue-type-dependent mechanism for the regulation of DPP4 in solid tumours. Further studies are necessary to identify the functional consequences of DPP4 processing and its potential prognostic or therapeutic value.

## 1. Introduction

Epithelial ovarian cancers (EOCs) remain the most lethal of gynaecological malignancies, and account for >80% of ovarian tumour diagnoses [1]. A lack of screening or early detection modalities means that most patients are diagnosed with advanced disease, and the majority of these relapse with chemoresistant disease within 18 months [2]. Despite efforts to develop targeted therapies, 5-year survival has remained at approximately 40% for the past 30 years [2]. There is an urgent and unmet need for improved diagnostic, prognostic and therapeutic tools to reduce mortality for ovarian cancer patients.

Dipeptidyl peptidase 4 (DPP4; CD26) is a member of the prolyl oligopeptidase serine protease family, which includes the related enzymes fibroblast activated protein (FAP), DPP8, and DPP9 [3]. Expressed by multiple cell types, DPP4 catalyses the cleavage of N-terminal *X-Pro* and *X-Ala* dipeptides from a variety of substrates to directly modify their bioactivities [4,5]. Indeed, a number of inflammatory mediators with central roles in immune suppression and tumour progression (e.g., CCL5, 11, 22; CXCL2, 6, 9–12) are potential targets for DPP4-directed cleavage [3,4,5]. DPP4 also non-enzymatically regulates adhesion via interaction with extracellular matrix (ECM) proteins (e.g., collagen, fibronectin), and is involved in signalling pathways through association with FAP and CXCR4 [6]. Accordingly, DPP4 expression is linked with tumorigenic behaviour in a variety of cancer types (reviewed in [6]).

Whilst DPP4 over-expression in ovarian cancer tissues is well established [5,7], both pro- and anti-tumour effects have been attributed to DPP4 [6] and in vitro analyses have generated significant conflict surrounding its functional significance. High DPP4 expression in ovarian cancer cells is correlated with an epithelial phenotype and reduced invasiveness [8,9]; conversely, DPP4 expression has also been associated with the enhanced migratory capacity and tumorigenic potential of ascites-derived cancer cells [9]. Similarly, whilst DPP4 expression in ovarian cancer cells varies according to cell type [10], studies disagree regarding in which cell types, and to what degree, DPP4 is expressed [9,10]. The functional role and prognostic significance of DPP4 expression in ovarian cancer thus remains unclear [6,10].

Within hypoxic tumour “nests” and poorly oxygenated ascites fluid [11], malignant ovarian cancer cells thrive and form multicellular aggregates (spheroids) that metastasize throughout the peritoneal cavity [11]. Hypoxia-inducible factor 1-alpha (HIF-1α), a master regulator of hypoxic response, is upregulated in ovarian tumour tissues and is correlated with chemo-resistance and decreased overall survival for ovarian cancer patients [12,13]. Some studies have demonstrated hypoxia-induced DPP4 expression in diverse cell types including smooth muscle cells and adipocytes [14,15,16]. Indeed, recent work in human preadipocytes showed that HIF-1α strongly induced DPP4 expression and potentially regulated its proteolytic release from the cell surface [15]. The strong expression of DPP4 in cancer tissues, together with high expression in cancer cells derived from poorly oxygenated ascites fluid [10,11], suggests that the tumour microenvironment can exert a distinct influence on DPP4 expression and function in vivo. However, the role of hypoxia on DPP4 expression in ovarian cancer has never been evaluated.

In this study we have investigated the role of hypoxic conditions on DPP4 expression and function in ovarian cancer cells in vitro. We provide evidence for the hypoxic regulation of DPP4 in ovarian cancer cells and identify a potential matrix metalloproteinase-mediated mechanism of DPP4 proteolysis from the cell surface. Importantly, whilst the mechanism of hypoxia-induced DPP4 regulation also occurs in non-malignant cells, our data shows that the enzymes regulating DPP4 function differ in malignancy and suggest complex regulation of DPP4 in tumour versus non-tumour tissues.

## 2. Results

### 2.1. DPP4 Expression is Upregulated by Hypoxia in Ovarian Cancer Cells

**Figure 1:**
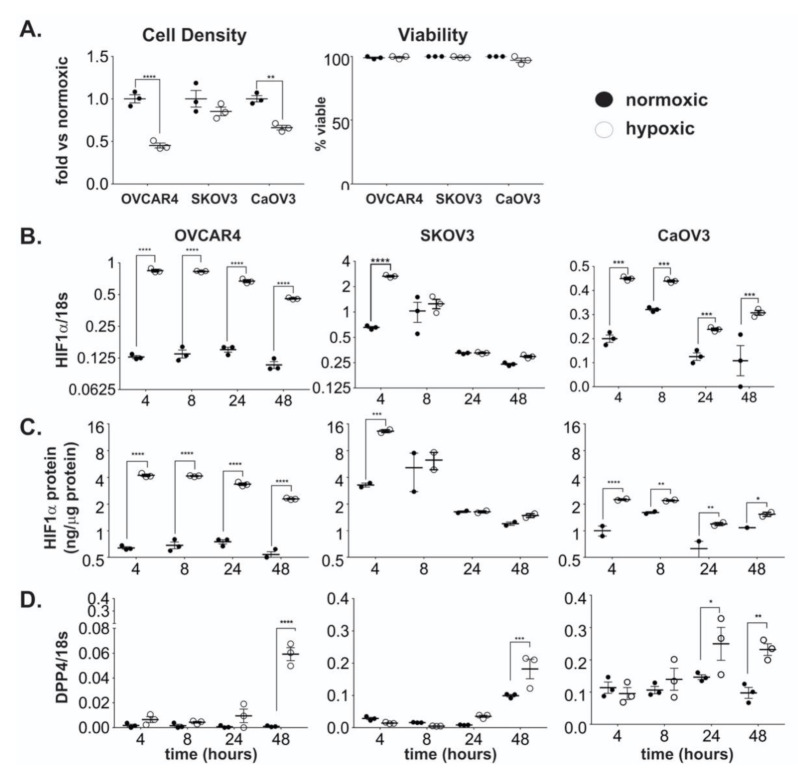
The effect of hypoxia on cell growth, HIF-1α and DPP4 expression in ovarian cancer cells. Ovarian cancer cell lines OVCAR4, SKOV3, and CaOV3 were seeded at ~10^6^ cells/25 cm^2^ flask and cultured under normoxic or hypoxic conditions for up to 48 h. (**A**) Cell density and viability at 48 h. (**B**) HIF-1α mRNA expression and (**C**) protein abundance were measured by qRT-PCR and ELISA respectively. (**D**) DPP4 mRNA expression was measured by qRT-PCR. Data represent the mean ± SD, *n* = 3/group. *p < 0.05; **p < 0.01; ***p < 0.001; ****p < 0.0001.

Several studies suggest that the in vitro and in vivo expression of DPP4 may be affected by hypoxia in a cell- and tissue-dependent manner [14,16,17]. To assess the effect of hypoxic growth on DPP4 expression and abundance in ovarian cancer, cell lines OVCAR4, CaOV3, and SKOV3 were cultured under either normoxic (20% O_2_) or hypoxic (2% O_2_) conditions for 48 h. Cells cultured under prolonged hypoxia reached a lower overall cell density, however, cell viability was unaffected (Figure 1A).

Samples were collected following 4, 8, 24, and 48 h of incubation and assessed for HIF-1α (to confirm induction of hypoxia) and DPP4 expression. In high grade serous cancer (HGSC) cell lines OVCAR4 and CaOV3 [18], *HIF-1α* mRNA expression was rapidly induced under hypoxia and remained elevated compared to normoxic levels over the incubation period (Figure 1B). Adenocarcinoma- derived SKOV3 line which forms clear cell tumours in vivo [18,19,20] displayed acute induction of *HIF-1α* mRNA after 4 h, which returned rapidly to a similar level to cells grown under normoxic conditions (Figure 1B). *HIF-1α* was notably more abundant in SKOV3 than OVCAR4 or CaOV3 cells, consistent with higher HIF-1α expression reported in clear cell ovarian tumours compared to other histotypes [21]. Corresponding protein analysis by ELISA demonstrated parallel increases over time of HIF-1α protein in all cell types (Figure 1C).

Each cell line was assessed for the induction of *DPP4* expression. In every case, *DPP4* mRNA increased in a time-dependent manner in cells grown under hypoxic conditions compared to those grown under normoxia (Figure 1D). By 48 h, all cells grown under hypoxia showed significantly elevated *DPP4* mRNA expression. Whilst lower cell densities were observed under hypoxic versus normoxic growth, the increase in *DPP4* expression was not associated with changes in cell viability after 48 h (Figure 1A). This incubation period was selected as cells that were cultured under hypoxia for longer than 48 h demonstrated decreased cell viability (data not shown). Together these results show *DPP4* mRNA expression is consistently upregulated under conditions of chronic hypoxic growth in ovarian cancer cell lines.

### 2.2. Chronic Hypoxia Induces DPP4 Shedding from the Surface of Ovarian Cancer Cells

**Figure 2:**
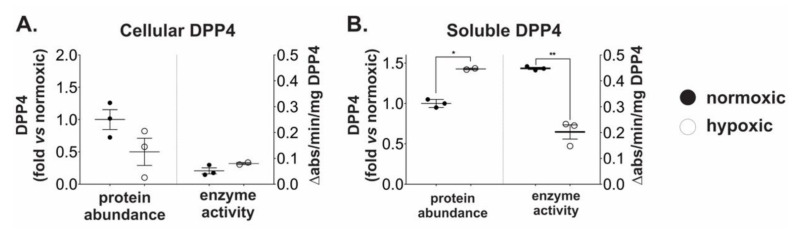
Hypoxia induces shedding of inactive DPP4 from ovarian cancer cells. OVCAR4 cells were cultured under normoxia or hypoxia for 48 h. ELISA for quantitation of DPP4 protein abundance and enzyme assays for DPP4 activity were performed on (**A**) cell lysates and (**B**) conditioned media. Enzyme activity is expressed as specific activity relative to the measured abundance of DPP4. The data represent the mean ± SD, *n* = 3/group. *p < 0.05; **p < 0.01.

Since OVCAR4 cells displayed the most robust hypoxic response amongst the three cell lines examined, they were used as a model to measure changes in DPP4 abundance and activity under hypoxia. Unexpectedly, despite the induction of *DPP4* mRNA expression there was no significant difference in DPP4 protein abundance in cells grown under hypoxic conditions (Figure 2A). Moreover, cellular DPP4 enzyme activity remained unchanged (Figure 2A) suggesting DPP4 abundance and enzyme activity in cells were maintained in a relatively steady state under hypoxic conditions.

DPP4 can exist in both cellular and secreted (sDPP4) forms, and it has been suggested that hypoxia may stimulate sDPP4 release from the cell surface [15]. We therefore also assessed DPP4 abundance and enzyme activity in conditioned media from cells grown under hypoxia. By contrast to cell lysates, conditioned media contained ~50% more sDPP4 protein following hypoxic growth (Figure 2B). Paradoxically, sDPP4 specific enzyme activity decreased under these conditions (Figure 2B). Western blotting was unable to confirm proteolysis of sDPP4 in the Fetal Bovine Serum (FBS)-supplemented media (see Supplementary Figure S1A); however, detection by ELISA argued against extensive degradation of sDPP4 following secretion. Together, these data indicate that cellular DPP4 abundance and activity is maintained in a steady state during hypoxia by the release of sDPP4 into the surrounding milieu. However, it also demonstrates that sDPP4 is likely inactivated either during or soon after release from ovarian cancer cells in vitro.

### 2.3. Chronic Hypoxic Growth Stimulates Matrix Metalloproteinase Expression and Alters DPP4 Release from Cells

**Figure 3:**
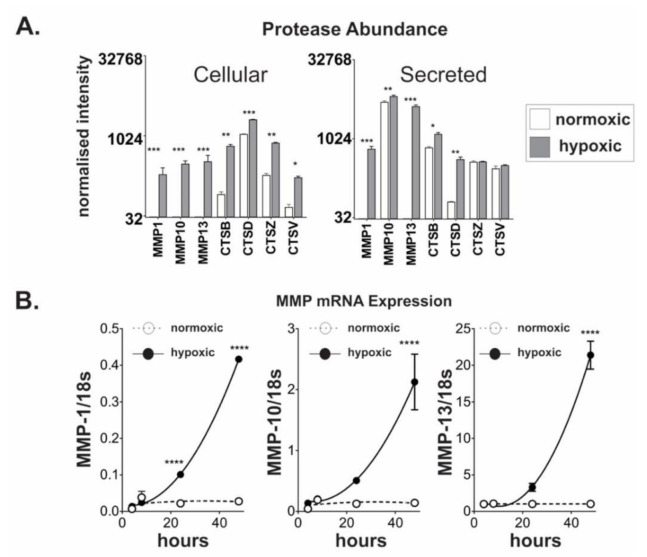
Hypoxia regulates protease expression in ovarian cancer cells. Cell lysates and conditioned media from OVCAR4 cells grown under normoxia and hypoxia were analysed for abundance of 27 different human proteases (Supplementary Table S1) using antibody arrays. (**A**) Normalized detection intensities for cellular and secreted proteases (MMP1, MMP10, MMP13, CTSB, CTSD, CTSZ, and CTSV) that were significantly different following hypoxic incubation of OVCAR4 cells. (**B**) Expression levels of *MMP1*, *MMP10*, and *MMP13* were validated by qRT-PCR. The data represent the mean ± SD, *n* = 3/group. *p < 0.05; **p < 0.01; ***p < 0.001; ****p < 0.0001.

Matrix metalloproteinases (MMPs) are well-characterized for their roles in ECM remodelling and are strongly associated with tumour invasion [22]. Based on recent data suggesting that MMPs may mediate shedding of DPP4 from smooth muscle cells [15], we hypothesized that MMPs may play a similar role in ovarian cancer cells. Cell lysates and conditioned media from OVCAR4 cells grown under normoxic or hypoxic conditions, were analysed for changes in the abundance of selected proteases (Figure 3A, Supplementary Table S1). Under hypoxic growth conditions, we observed significant increases in the cellular abundance of MMPs 1, 10, and 13, and substantially increased secreted levels of these MMPs (Figure 3A and Supplementary Figure S1B). Likewise, the cellular abundance of cathepsins CTSB, CTSD, CTSV, and CTSZ was also increased under hypoxia; however, extracellular CTSV and CTSZ were unaltered, and only moderate changes in extracellular CTSB and CTSD were detected (Figure 3A).

The changes in MMPs 1, 10, and 13 were further explored by qRT-PCR. In each case, the gene expression of *MMP1, MMP10*, and *MMP13* was substantially increased following prolonged exposure to hypoxia (Figure 3B). In addition, *DPP4* expression was strongly correlated (Pearson correlation) with *MMP1* (*r* = 0.99, *p* = 0.007), *MMP10* (*r* = 0.99, *p* = 0.011), and *MMP13* (*r* = 0.98, *p* = 0.024) expression in cells grown under hypoxic, but not normoxic, conditions.

**Figure 4:**
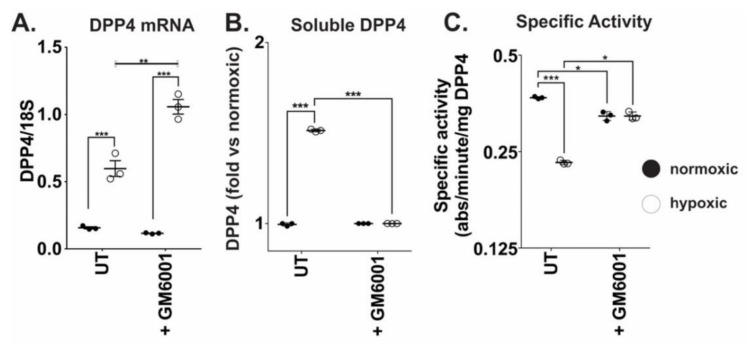
Matrix metalloproteinases (MMP) activity influences DPP4 expression and release in ovarian cancer cells. OVCAR4 cells were treated with the pan-MMP inhibitor GM6001 (25 µM) and cultured under normoxia or hypoxia for 48 h. Cells were harvested and analysed for (**A**) DPP4 mRNA expression; and conditioned media were collected and analysed for (**B**) soluble DPP4 protein abundance and (**C**) DPP4 enzyme activity. The data represent the mean ± SD, *n* = 3/group. *p < 0.05; **p < 0.01; ***p < 0.001.

We next used the pan-MMP inhibitor GM6001 [23] to broadly assess the role of MMP enzyme activity in sDPP4 release from OVCAR4 cells. Cells treated with GM6001 remained sensitive to hypoxia and showed an increase of *DPP4* mRNA expression at 48 h (Figure 4A). Global MMP activity was therefore not required for DPP4 expression. However, compared to untreated cells, hypoxia failed to induce sDPP4 secretion from GM6001 treated cells or stimulate the previously observed reduction in sDPP4 activity (Figure 4C), confirming that sDPP4 release from cells was influenced by MMP activity in vitro.

### 2.4. MMP10 and MMP13 Mediate DPP4 Expression and Release from Cancer Cells

**Figure 5:**
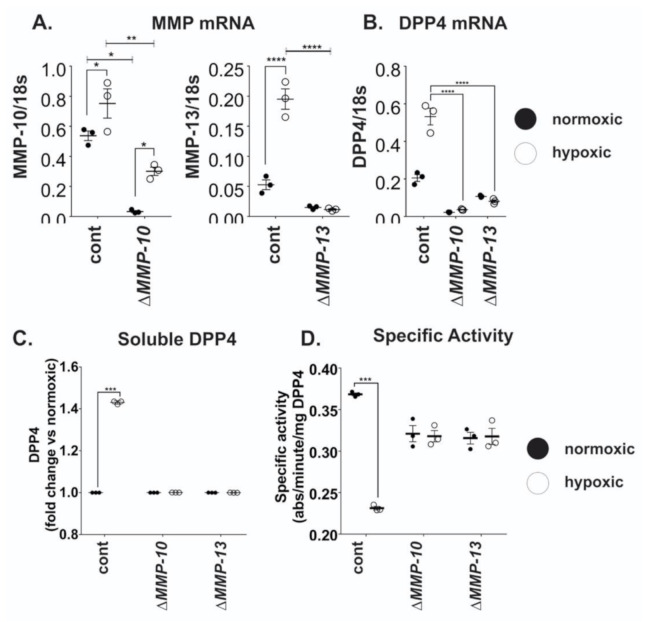
MMP10 and MMP13 affect DPP4 expression and release from ovarian cancer cells. OVCAR4 cells with stable shRNA knockdown of *MMP10* or *MMP13* were cultured under normoxia or hypoxia for 48 h. Total RNA was extracted, and qRT-PCR performed to confirm (**A**) knockdown of *MMP10* and *MMP13* relative to controls, and (**B**) examine the effects of MMP10 and MMP13 on *DPP4* mRNA expression. DPP4 ELISA and enzyme assay were performed on conditioned media of cells to assess changes in (**C**) sDPP4 release and (**D**) specific activity induced by the downregulation of *MMP10* and *MMP13*. The data represent the mean ± SD, *n* = 3/group. *p < 0.05; **p < 0.01; ***p < 0.001; ****p < 0.0001.

To more deeply explore the relationship between DPP4 and MMPs, shRNA was used to individually knock down the expression of *MMP1, MMP10*, and *MMP13* in OVCAR4 cells. Efficient knock down of both *MMP10* and *MMP13* was confirmed by qRT-PCR (Figure 5A); *MMP1* knock down (KD) was unsuccessful (not shown) and was not examined further. When challenged with hypoxia, *MMP10* mRNA expression was reduced by ~60% in *MMP10-*KD cells (Figure 5A); whilst knock-down of *MMP13* resulted in complete inhibition of *MMP13* expression under both normoxic and hypoxic conditions (Figure 5A).

Compared to control cells, hypoxia failed to induce DPP4 expression in either *MMP10-*KD or *MMP13-*KD cells (Figure 5B). Consistent with the lack of *DPP4* induction following MMP knock-down, neither *MMP10-*KD nor *MMP13-*KD cells displayed any difference in DPP4 abundance or activity under hypoxia (Figure 5C,D). Control cells expressing the scrambled shRNA control responded as previously observed. Together with the results of pan-MMP inhibition (above), these data suggest that DPP4 shedding from the ovarian cancer cell surface is mediated by MMP activity, whilst DPP4 mRNA expression is influenced by both MMP10 and MMP13 at the transcriptional level, suggesting the involvement of a more complex regulatory mechanism.

## 3. Discussion

A key factor contributing to ovarian cancer progression is the hypoxic environment within the tumour mass and peritoneal ascites fluid. The expression of HIF-1α as a marker of hypoxic response is strongly correlated with chemo-resistance and is an independent predictor of poor prognosis for EOC patients [12,13]. Several studies have linked the induction of DPP4 in response to hypoxia in vitro and in vivo [15,24]. However, DPP4 abundance and activity vary between different cancer types, and exhibit complex patterns of regulation [25]. Neither the expression nor function of DPP4 in ovarian cancer cells have previously been examined under conditions of hypoxic growth that mimic the tumour microenvironment.

When cultured under conditions of low atmospheric oxygen for prolonged periods, we identified a significant and sustained increase in *DPP4* expression in all ovarian cancer cell lines examined. Whilst there is evidence proposing DPP4 as a marker of HIF-1α induction [14], our data suggested that DPP4 expression was largely independent of HIF-1α status; however, all cell lines tested constitutively expressed HIF-1α, potentially masking any specific or HIF-1α dependent effects. Typical of a housekeeping gene, DPP4 can be regulated in a cell-type-specific manner [26,27,28] by transcription factors including Stat1α and hepatocyte nuclear factor-1 (HNF-1), specific physiological stimuli (e.g., secreted cytokines) and post-translational modifications (e.g., glycosylation [29]), all of which can be affected by the hypoxic tumour microenvironment [28]. Our data evidences a previously undescribed mechanism of DPP4 regulation in ovarian cancer cells mediated according to oxygen status, either directly via HIF-1α modulation or indirectly via its downstream targets.

An unexpected and novel finding was the active shedding of inactivated DPP4 from cancer cells under hypoxic growth, which was mediated in part by an increase in MMP activity. There is conflicting evidence across multiple cancer types regarding the regulation of DPP4 expression and activity, however, prior studies have failed to consider the hypoxic tumour microenvironment that is common to many solid cancers. Our discovery of the uncoupling of DPP4 expression from enzyme activity in cancer cells provides a plausible explanation for this previously unclear role of DPP4 in various tumours, including ovarian, colorectal, and potentially other cancers. In non-malignant cells such as smooth muscle and adipocytes, hypoxia-induced DPP4 shedding is mediated by increased activity of MMPs 1, −2, and −14 [15]. Our data suggests that the mechanism of DPP4 release from cancer cells is likely to be different, involving proteolytic release and inactivation mediated by at least MMP10 and MMP13, which directly supports recent evidence that hypoxia promotes ovarian cancer cell invasion via an MMP-13 mediated mechanism [30]. By analogy, DPP4 is shed from the surface of CD4+ T cells by kallikrein-related peptidase 5 (KLK5) [31], and in hepatoma cells Serpin B3 induces overexpression of inactive DPP4 [32]. The regulation of DPP4 shedding from the cell surface, and the activation status of sDPP4, is thus a cell-type dependent phenomenon. Whether DPP4 shedding in ovarian cancer cells is directly mediated by MMP activity, via MMP-dependent activation of pro-enzymes, or by a more complex interrelationship with other protease systems [33] requires further investigation. In addition, the possibility of other mechanisms of sDPP4 release including vesicle-mediated trafficking and factors which influence surface expression such as genetic heterogeneity should also be considered in future studies.

Elevated levels of circulating sDPP4 have been suggested as a prognostic biomarker in colorectal cancer, where they are associated with reduced overall and progression free survival [34]. Interestingly, decreased sDPP4 enzyme activity in colorectal cancer relative to healthy controls is also associated with poor prognosis [35], and a similar decrease in activity occurs in patients with melanoma [36]. These findings are consistent with our data, where increased extracellular DPP4 abundance was accompanied by decreased activity. Whilst altered DPP4 glycosylation or phosphorylation [37] could also influence the reliability of sDPP4 measurement, it is likely that enzymatic processing and inactivation of DPP4 plays a role in at least some solid tumour types to influence both abundance and activity status. The measurement of specific, proteolytic DPP4 fragments shed by cancer cells may thus be more relevant as a biomarker of disease status.

Although DPP4 has important functions in metabolism, recent data also support its key roles in immunity and inflammation [4,7,38]. DPP4 can regulate the bioactivity of several chemokines to influence immune and inflammatory responses critical to cancer progression [7,25,39]. For example, suppression or down-regulation of DPP4 promotes prostate cancer progression via reduced degradation of its substrate CXCL12, leading to enhanced signalling via CXCR4 and increased invasion and metastatic spread to peripheral organs in vivo [40,41]. Similarly, reduced DPP4 activity in poorly differentiated gliomas [42] and an associated decrease in Substance P processing is linked to the loss of cell growth inhibition via reduced calcium signalling [43]. Altered DPP4 function is also postulated as an underlying mechanism in the poor response of some high grade ovarian cancers [7], and the in vitro and in vivo interactions between DPP4 and specific cytokines are established as an important mechanistic contribution in different cancer types [7,38]. The apparent cancer-specific incidence of DPP4 inactivation thus suggests the potential of inhibiting DPP4 in tailored therapeutic interventions to improve anti-tumour immunity. Indeed, DPP4 inhibition using the anti-diabetes drug Sitagliptin has been associated with reduced incidence of breast cancer in diabetes patients [44], and has an immune-mediated protective effect in several preclinical models [38,45,46]. For example, Sitagliptin treatment improves anti-tumour responses via enhanced CCL11-mediated eosinophil migration in hepatocellular carcinoma mouse models [45], and promotes CXCL9- and CXCL10-mediated dendritic cell trafficking to melanoma tumours [46].

Cell migration and invasion are accompanied by proteolytic degradation of the ECM, in which MMPs, Kallikreins, Cathepsins, and DPP4 all have established roles. In particular, the DPP4-FAP heterodimer localizes at the cell surface as an enzymatically active complex and is correlated with the upregulation of MMPs [3,47]. DPP4 enzyme activity also promotes cell migration and adhesion in cervical cancer lines [48], and its inhibition is associated with decreased invasion, migration, and colony formation capacity in thyroid cancer cells [49]. It is unclear, however, how DPP4 inactivation might influence invasion in the poorly oxygenated peritoneal environment and ascites fluid present in metastatic ovarian cancers [11]. DPP4 expression has been associated with tumour initiating cell populations in several tumour types [50,51], and ovarian cancer spheroids (free-floating organoids with a hypoxic core) found in ascites fluid contain putative ovarian cancer stem cells [52]. The alterations in DPP4 abundance and activity observed under hypoxic conditions may therefore be related to a transition state between transcriptionally plastic cell phenotypes, required to preserve or maintain cancer cell populations during hypoxic stress.

## 4. Materials and Methods

### 4.1. Cell Culture

The human ovarian cancer cell lines SKOV3 (RRID: CVCL_0532) and CaOV3 (RRID: CVCL_0201) were purchased from the ATCC. The OVCAR4 (RRID: CVCL_1627) cell line was a kind gift from Professor David Bowtell (Peter MacCallum Cancer Centre, Melbourne). All cell lines have been authenticated using STR profiling within the last three years. SKOV3, CaOV3, and OVCAR4 cells were maintained in Dulbecco’s Modified Eagle’s Medium (DMEM)/Nutrient Mixture F-12 Ham (Sigma-Aldrich, St. Louis, MO, USA), DMEM (Sigma-Aldrich, St. Louis, MO, USA) or RPMI-1640 Medium (Sigma-Aldrich, St. Louis, MO, USA), respectively; supplemented with 10% Fetal Bovine Serum (FBS) (Thermo Fisher Scientific, Waltham, MA, USA) and 1% penicillin/streptomycin (p/s; Thermo Fisher Scientific, Waltham, MA, USA). Cells were incubated at 37 °C under either normoxia (20% O_2_, 5% CO_2_) or hypoxia (2% O_2_, 5% CO_2,_ 150 µM CoCl_2_; BioSpherix hypoxia suite) as indicated in the text. Cell counts and viability were determined using Trypan Blue cell viability dye and the Countess II FL automated cell counter (Thermo Fisher Scientific, Waltham, MA, USA). All lines were validated mycoplasma-negative.

### 4.2. Quantitative Real-Time PCR (qRT-PCR)

Total RNA was extracted from cells using Ultraspec^®^ RNA reagent (Fisher Biotec Australia, Wembley, Australia) according to the manufacturer’s instructions. cDNA was synthesized by reverse transcription from 1 µg RNA using the Tetro cDNA synthesis kit (Bioline, London, UK). For quantitative real-time PCR (qRT-PCR) analysis of specific genes, sense and antisense oligonucleotide primers were designed against published human sequences and verified as previously described [53]. Primer pairs for each gene are listed in Table 1. Real-time PCR samples were prepared to a final volume of 10 µl using the Applied Biosystems *Power* SYBR™ Green PCR Master Mix. Quantitative real-time PCR was completed as previously described [53] using the QuantStudio™ 6 Flex Real-Time PCR System (Applied Biosystems, Foster City, CA, USA) with all reactions performed in triplicate. Yields were converted to femtograms based on the standard curve for each PCR product, and the resultant mRNA levels were normalized to the *18S* mRNA level per sample (averaged over 3 replicates).

### 4.3. Enzyme Linked Immunosorbent Assay (ELISA)

Protein quantitation in total cell lysate or cell culture supernatant were performed using commercially available ELISA kits (Human HIF-1-alpha; ab171577 and Human CD26; ab11951, Abcam, Cambridge, UK) as per the manufacturer’s instructions. CD26 ELISA was performed using 10 µg of protein from cell lysate or concentrated cell culture supernatant. HIF-1-alpha ELISA was performed using 5 µg of protein lysate. Absorbance was measured using a FLUOstar^®^ Omega microplate reader (BMG Labtech, Mornington, Australia).

### 4.4. DPP4 Enzyme Activity Assays

DPP4 enzymatic activity in cell lysates was measured as described [5] using 15 µg of total protein lysate per well. Substrate hydrolysis was measured every 10 min for 3 h using a Cytation™ 3 Multi-Mode Reader (BioTek, Winooksi, VT, USA) at 37 °C. Absorbance readings at 570 nm were subtracted from readings at 405 nm to account for the optical interference from cell culture media. All samples were measured in triplicate.

For secreted DPP4 enzymatic activity, a more sensitive fluorogenic assay was used. In each case a 2 mM solution of the fluorogenic DPP4 substrate Gly-Pro-7-amido-4-methylcoumarin (Sigma-Aldrich, St. Louis, MO, USA) in Tris-EDTA buffer with 10% methanol was added to each well of a black 96-well plate containing 15 µg of concentrated cell culture supernatant from an initial volume of 1 mL. Each sample was assayed in triplicate and substrate hydrolysis was determined by measuring fluorescence at 355_ex_/450_em_ every 10 min for 3 h using the Cytation™ 3 Multi-Mode Reader at 37 °C.

### 4.5. Protease Arrays

Relative quantitative measurement of 40 unique proteases in cell lysates and cell culture media was determined using commercially available antibody arrays (Human MMP antibody array, ab197453 Abcam, Cambridge, UK; and Proteome Profiler^TM^ Array, ARY021 R&D Systems, Minneapolis, MN, USA) as per the manufacturer’s instructions. Each array was probed using 50 µg of total protein from either cell lysate or concentrated cell culture supernatant. Arrays were visualized using either a Typhoon Variable Mode Imager (Molecular Dynamics, Sunnyvale, CA, USA) equipped with a Cy3 wavelength filter (Human MMP array) or a ChemiDoc™ XRS+ System (BioRad, Hercules, CA, USA) to detect chemiluminescence (Proteome Profiler array). Signal intensities were determined using Fiji software v1.0 [54,55], and replicate values were individually normalized against a reference array.

### 4.6. Targeted Knockdown of Selected MMPs Using shRNA

To inhibit human *MMP10* and *MMP13* expression in vitro, we designed fold-back stem-loop shRNA structures using the design recommendations by System Biosciences (Palo Alto, CA, USA). Inhibitory stem-loops comprised a 21-bp sense strand (obtained from the Sigma MISSION^®^ shRNA database) identical to the coding region of the target gene, a 12-bp loop (CTTCCTGTCAGA) and a 21-bp antisense strand followed by an RNA polymerase III terminator sequence (TTTTT). Restriction sites for *Bam*HI and *Eco*RI were incorporated at the 5′ and 3′ end of the shRNA, respectively, for cloning into the pSIF-H1-Puro vector (System Biosciences, Palo Alto, CA, USA). Construct identity was confirmed by sequencing analysis. For MMP knockdown pSIF-H1-Puro-*MMP10* or *-MMP13* vectors were transfected into the OVCAR4 cell line using Lipofectamine^®^ 2000 (Invitrogen, Carlsbad, CA, USA) according to the manufacturer’s instructions. Positive transfectants were selected using Puromycin (1 µg/mL; Sigma-Aldrich, St. Louis, MO, USA). Total cellular RNA was extracted and MMP expression was assessed by qRT-PCR (as above) using the following primers; *MMP10* F: 5′**-**CACAGTTTGGCTCATGCCTA-3′, *MMP10* R: 5′**-**TGCCATTCACATCATCTTGC-3′; *MMP13* F: 5′**-**TTGAGCTGGACTCATTGTCG-3′, MMP13 R: 5′**-**CTCAGTCATGGAGCTTGCTG-3′.

### 4.7. Statistical Analysis

All statistical analyses were performed using GraphPad Prismv7.0b (GraphPad Software Inc., San Diego, CA, USA). Significance between groups was determined by two-way ANOVA and Bonferroni’s multiple comparisons test, or by paired *t*-test. Spearman’s correlation was used to measure the strength of relationships between variables. All data are presented as mean ± SD. Results with a *p*-value ≤ 0.05 were considered significant.

## 5. Conclusions

The current study provides the first insights into the potential influence of the hypoxic microenvironment on DPP4 expression and function in ovarian cancers, and we have shown for the first time that MMP enzyme activity is necessary for DPP4 shedding from ovarian cancer cells, suggesting similar mechanisms may exist in other cancers where DPP4 plays a role. The discovery of a link between hypoxia, DPP4 expression and MMP-dependent shedding offers a much-needed new mechanistic understanding into potentially contradictory findings in the literature on DPP4 expression and its prognostic utility in ovarian cancer. Our data demonstrates a highly regulated relationship between DPP4 function and expression and hypoxia-induced proteases in the tumour microenvironment and suggests further studies are needed to elucidate the complex role of DPP4 in epithelial ovarian cancers and other solid tumours.

## Figures and Tables

**Table 1:** Human primer sequences for real-time PCR.

Gene	Accession Number	Primer Sequences 5′–3′	
		Forward	Reverse
***18S***	NR_003286.4	GTAACCCGTTGAACCCCATT	CCATCCAATCGGTAGTAGCG
***HIF-1α***	NM_001530	CAGCTATTTGCGTGTGAGGA	CCTCATGGTCACATGGATGA
***DPP4***	NM_001935	ATGCCAGGAGGAAGGAATCT	TATAGAGGGGCAGACCAGGA
***MMP1***	NM_002421	GGACAACTCTCCTTTTGATGGA	CAAAGCCCCGATATCAGTAGAA
***MMP10***	NM_002425	CACAGTTTGGCTCATGCCTA	TGCCATTCACATCATCTTGC
***MMP13***	NM_002427	GACCCTGGAGCACTCATGTT	TCCTCGGAGACTGGTAATGG

## Supplementary Materials

The following are available online at https://www.mdpi.com/1422-0067/21/21/8110/s1.
