# Supplementary material for: Hypoxia Regulates DPP4 Expression, Proteolytic Inactivation, and Shedding from Ovarian Cancer Cells
Source: Int J Mol Sci. 2020 Oct 30;21(21):8110. doi: 10.3390/ijms21218110 (PMC7672561; doi:10.3390/ijms21218110)

**Figure S1.** **Protease expression in the culture media of ovarian cancer cells.** Conditioned media from OVCAR4 cells grown under normoxia and hypoxia were analysed for abundance of several different human proteases (Supplementary Table S1) using antibody arrays. Raw images indicating (A) CD26/DPP4 and (B) MMP-10 and MMP-13 protein abundance in conditioned media of OVCAR4 cells.


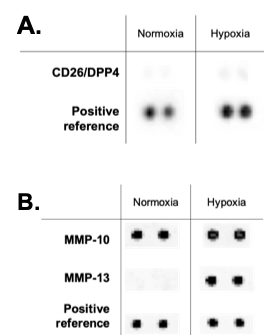

Supplement: Supplementary file 1 [file ijms-21-08110-s001.zip › IJMS 2020_SuppFigure1.docx]
