# Supplementary material for: Hypoxia Regulates DPP4 Expression, Proteolytic Inactivation, and Shedding from Ovarian Cancer Cells
Source: Int J Mol Sci. 2020 Oct 30;21(21):8110. doi: 10.3390/ijms21218110 (PMC7672561; doi:10.3390/ijms21218110)
Supplement: Supplementary file 1 [file ijms-21-08110-s001.zip › IJMS 2020_SuppTable1.docx]

**Table S1.** List of human protease targets analysed in the cell lysates and culture media of OVCAR4 cells (MMP array; Abcam ab197453, Protease array; R&D ARY021).

| **MMP array** | **Protease Array** | | |
| --- | --- | --- | --- |
| MMP-1 | ADAM8 | Cathepsin X/Z/P | MMP-3 |
| MMP-2 | ADAM9 | DPPIV/CD26 | MMP-7 |
| MMP-3 | ADAMTS1 | Kallikrein 3/PSA | MMP-8 |
| MMP-8 | ADAMTS13 | Kallikrein 5 | MMP-9 |
| MMP-9 | Cathepsin A | Kallikrein 6 | MMP-12 |
| MMP-10 | Cathepsin B | Kallikrein 7 | MMP-13 |
| MMP-13 | Cathepsin C | Kallikrein 10 | Neprilysin/CD10 |
| TIMP-1 | Cathepsin D | Kallikrein 11 | Presenilin-1 |
| TIMP-2 | Cathepsin E | Kallikrein 13 | Proprotein Convertase 9 |
| TIMP4 | Cathepsin L | MMP-1 | Proteinase 3 |
|  | Cathepsin S | MMP-2 | uPA/Urokinase |
|  | Cathepsin V |  |  |
